# Identification and verification of international neuroblastoma staging system (INSS) stage-related genes as potential biomarkers for neuroblastoma prognostic models

**DOI:** 10.3389/fcell.2025.1502380

**Published:** 2025-04-15

**Authors:** Can Qi, Ziwei Zhao, Yanwei Qi, Yun Zhou, Fang Yue, Huizhong Niu, Guochen Duan, Zhiyong Zhong, Le Wang

**Affiliations:** ^1^ Institute of Pediatric Research, Children’s Hospital of Hebei Province, Shijiazhuang, Hebei, China; ^2^ Department of Thoracic Surgery, Hebei General Hospital, Shijiazhuang, Hebei, China

**Keywords:** neuroblastoma, prognosis, biomarkers, INSS stage, pediatric

## Abstract

**Background:**

Neuroblastoma (NB), one of the most common malignant extracranial solid tumors in children, is highly invasive and lethal with limited treatment efficacy. This study aimed to establish a prognostic model of advanced-stage NB.

**Methods:**

Differentially expressed genes were screened and validated using two training datasets and one validation dataset from the Therapeutically Applicable Research to Generate Effective Treatments and Gene Expression Omnibus databases. Protein–protein interaction networks were developed using the MCode plug-in, and the top three key clusters were used to produce candidate genes. We performed gene set enrichment analysis (GSEA), gene ontology analysis (GO), Kyoto Encyclopedia of Genes and Genomes (KEGG) analysis, immune cell infiltration, and drug sensitivity analysis to further understand the functions of these candidate genes. Kaplan–Meier (K–M) and receiver operating characteristic (ROC) curves were used to check their prognosis value. Real-time quantitative polymerase chain reaction (qPCR), Western blot (WB), and immunohistochemistry (IHC) were employed to verify the mRNA and protein levels in clinical samples.

**Results:**

A total of 699 differentially expressed genes were identified, including 294 upregulated and 405 downregulated genes. *CNR1, PRKACB, CDKN3*, and *PCLAF* were found to significantly affect the overall survival and event-free survival of neuroblastoma patients and were positively correlated with the INSS advanced stages. The functional analysis of these four genes revealed their cancer-promoting effects and correlations with immune-inflammatory, cell cycle, and p53 signaling pathways. After stratifying patients using the established model containing the above four genes, significantly different patterns were observed in terms of infiltrating immune cell proportion, drug sensitivity, and the expression of immune checkpoints. Finally, both the mRNA and protein expression verification assays demonstrated that the *CDKN3* and *PCLAF* were upregulated, while the *PRKACB* was downregulated in advanced-stage neuroblastoma tissue samples.

**Conclusion:**

The model containing *CNR1, PRKACB, CDKN3*, and *PCLAF* can serve as a new prognostic biomarker for predicting the prognosis of patients with neuroblastoma. Findings on immune cell infiltration and immune checkpoints provide novel insights for the immunotherapy of neuroblastoma.

## Background

Neuroblastoma (NB) is an embryonal tumor with extensive molecular heterogeneity in its biological and clinical presentation (Matthay et al., 2016). Although some NBs resolve spontaneously without treatment, approximately 50% of patients are classified as high risk on diagnosis, and only 50% survive for 5 years (Shohet et al., 2021). Accounting for 7%–10% of all childhood cancers, NB contributes 15% of cancer-related deaths (Irwin and Park, 2015). The pathogenesis of NB is reportedly associated with a number of genetic aberrations in genes regulating the cell cycle, cell proliferation, and programmed cell death (Salemi et al., 2022). Despite the emergence of many new treatment modalities for NB, such as radioimmunotherapy and molecular targeting therapies (Feng et al., 2023; Zafar et al., 2021), the estimated 5-year survival rates for patients with high-risk NB are approximately 50% (Shohet et al., 2021). According to the Children’s Oncology Group guidelines, NB can be divided into four International Neuroblastoma Staging System (INSS) stages (Brodeur et al., 1993). INSS is a postsurgical staging system that uses tumor location in relation to the midline structures, lymph node status, and the extent of previous surgical resection to define the tumor status. Bone marrow assessment and radiographic studies are used to detect metastases. Stage 4 or 4S determination depends on other clinical details (Naranjo et al., 2018).

Owing to the key role of INSS staging in assessing disease severity and prognosis, researchers continue to explore prognostic biomarkers associated with the INSS stage. MYCN gene amplification is more observed in neuroblastomas with higher INSS stages (Stages 3 and 4) (Hansen et al., 2017). Significantly higher TERT expression has been detected in NB samples with INSS Stage 4 than in samples with earlier stages (Akter and Kamijo, 2021). Differences in the 1p/11q (Raitio et al., 2021) and serum inflammatory factors (Zheng et al., 2020) were also identified by the stratification based on INSS staging. However, these studies either did not use transcriptomics and bioinformatics to screen genes at a hierarchical level, as in Secomandi et al., who compared only one gene, the lysosomal protease cathepsin D (Secomandi et al., 2022), or did not use clinically derived specimens for expression validation (Fan et al., 2020). Therefore, identifying specific markers for diagnosis and treatment is of vital importance for improving the prognosis of children with advanced INSS stage of NB.

Over the last few years, high-throughput sequencing and gene microarray technologies have been applied in many medical fields to facilitate the classification of tumors based on histological and clinical data, cancer-related genes, and biological pathways (Satam et al., 2023). Moreover, emerging bioinformatics based on gene expression has recently become an effective method for systematically screening tumor-related genes and exploring the related molecular mechanisms (Hernandez-Lemus et al., 2019). The early detection and prognosis of many diseases depend on biomarkers that can mark changes in the structure or function of systems, organs, tissues, cells, and subcells (Shawraba et al., 2021). Biomarkers can be used to classify NB into different groups, facilitating NB treatment and prognosis (Trigg et al., 2019). Several studies have discussed the prognostic role of biomarkers (Galardi et al., 2018; Glembocki and Somers, 2024; Liu et al., 2022); however, the clinical use of these biomarkers requires clinical evaluation. In this study, we screened patients with NB using the Therapeutically Applicable Research to Generate Effective Treatments (TARGET) and Gene Expression Omnibus (GEO) databases for potential biomarkers of survival prognosis. A novel approach for the prognostic diagnosis of NB was proposed. We identify markers of advanced-stage NB, explore their role in prognosis, and highlight the value of their implementation in a clinical setting. To effectively manage advanced-stage NB, the detection and analysis of prognostic biomarkers can play a vital role in refined risk assessment and the development of targeted therapeutic regimens.

## Materials and methods

### Data source

Training set 1 related to NB patients captured from the Therapeutically Applicable Research to Generate Effective Treatments (TARGET) database (https://ocg.cancer.gov/programs/target), called TARGET-NB. This set included clinical information and survival information for 152 patients with advanced stages of NB and was utilized for the prognostic model. From the Gene Expression Omnibus (GEO) database (http://www.ncbi.nlm.nih.gov/geo/), GSE73517 (GPL16876) was downloaded as training set 2 and utilized for differential expression analysis. The training set consisted of 105 samples; 20 tissues from patients with stages 1–2 of NB were selected as control samples (10 and 9 patients in stages 1 and 2, respectively), and 65 tissues from patients with advanced stages of NB (10 and 55 patients in stage 3 and 4, respectively). Stage 4S was not involved in this study. The validation set GSE62564 (GPL5175) contains 498 samples accompanied by survival information for validation of the prognostic model.

### Differential expression analysis

For choosing differentially expressed genes (DEGs) in stages 1–2 and 3–4 of NB patients, differential expression was undertaken based on the stages in training set 2 using R package limma version 3.54.0 (Ritchie et al., 2015). Screening was performed with threshold |log_2_fold-change (FC)| > 1, *adj. p* < 0.05. Using the R package ClusterProfiler version 4.2.2 (Wu T. et al., 2021), the acquired DEGs were analyzed for gene ontology (GO) and Kyoto Encyclopedia of Genes and Genomes (KEGG) with *p adj* < 0.05 to probe their possible roles. Furthermore, DEGs were submitted into the STRING database, with interaction = 0.7, and a protein–protein-interaction (PPI) network was developed. Subsequently, key clusters in PPI were analyzed by plug-in MCode in Cytoscape, and the top 3 clusters were selected by K-core = 2, degree cutoff = 2, max depth = 100, and node score cutoff = 0.2. The genes in these three clusters acted as candidate genes.

### Prognostic risk model

With the use of the coxph function in survival package version 3.3–5, univariate Cox regression was performed to select for genes linked to prognosis (HR ≠ 1, *p* < 0.05). A proportional hazards (PH) assumption was then tested on these genes, and a *p*-value greater than 0.05 indicated the genes did not influence each other. The least absolute shrinkage and selection operator (LASSO) regression analysis was fitted to the genes obtained from univariate Cox using the R package glmnet version 4.1-4 (Friedman et al., 2010). After 10-fold cross validation, the genes whose final regression coefficients were penalized to 0 were used as prognostic genes. A risk model was built with the expression and coefficients of the genes obtained from LASSO. The formula for this risk score was as follows:

$$riskscore=\sum_{i=1}^{n}\left({coef}_{i}*x_{i}\right).$$

The coef and x represented coefficients and relative expression levels of prognostic genes, respectively. A risk formula was adopted to calculate risk scores for patients with NB, and patients were sorted into high-/low-risk cohorts based on median risk scores to demonstrate score distribution and survival status in training set 1. Survival difference for patients from both risk cohorts was then analyzed using Kaplan–Meier (K–M) curves (*p* < 0.05). The performance of the risk model in predicting NB patient survival was also assessed with the help of receiver operating characteristic (ROC) curves. The risk model was then validated with the validation set using the same methodology.

### Independent prognostic analysis and nomogram construction

To assess the clinical applicability of the risk model, univariate and multivariate Cox regression analyses and Proportional Hazards tests were employed on risk scores and clinical characteristics based on training set 1 to screen the independent prognostic factors. With the rms package version 6.5-0, a nomogram was constructed depending on independent prognostic factors to predict survival for patients with NB. Calibration, ROC, and decision curve analysis (DCA) curves were plotted to evaluate the nomogram.

### Analysis of correlation between risk scores and clinical characteristics

For the purpose of exploring the relationship between risk scores and clinicopathological characteristics of NB patients, the distribution of risk score grades across different characteristics was demonstrated in training set 1. The differences among risk scores in different subgroups of clinical and molecular characteristics were confirmed.

### Functional enrichment analysis

With a view to understanding the biological pathways for risk cohorts, inter-cohort difference was analyzed via DESeq2 package version 1.38.3 (Love et al., 2014) in all the samples of training set 1, and log_2_FC was calculated and ranked from the largest to the smallest and then subjected to gene set enrichment analysis (GSEA). The reference gene set was KEGG (c2.cp.kegg.v2023.1.Hs.symbols.gmt). In addition, gene set variation analysis (GSVA) scores were calculated for training set 1 in the KEGG pathway using the GSVA package (v 1.42.0) (Hanzelmann et al., 2013), and the T-test was used to compare GSVA scores for prognostic genes in the early- and advanced-stage NB groups (*p* < 0.05).

### Immune microenvironment and drug sensitivity analysis

To assess immune cell infiltration associated with risk cohorts, patients with *p*-values <0.05 and incomplete cell content of 0 in samples of training set 1 were screened for the study using CIBERSORT analysis. The proportions of 22 immune cells in each NB patient were further counted. Immune cell differences were compared between risk cohorts. In addition, eight immune checkpoints were analyzed in the inner cohort of training set 1 (Wu J. et al., 2021). To evaluate drug sensitivity, the half-maximal inhibitory concentration (IC_50_) of each drug in each tumor sample in training set 1 was counted via oncoPredict. Differences in the IC50 of drugs were compared inter-cohorts, and the top 20 drugs were selected for presentation.

### Clinical sample collection

This study was approved by the Ethics Committee of Hebei Children’s Hospital (No. 2020-14), and written informed consent was obtained from each patient or guardian. Clinical data of children with neuroblastoma admitted to Children’s Hospital of Hebei (CHH) from March to December 2023 and their fresh neuroblastoma tissues obtained during tumor resection were collected and frozen for spare use; all of the above clinical tissue samples were obtained from the first time the children were admitted to the hospital, and they had not received any relevant treatment prior to surgery.

### Quantitative real-time PCR for mRNA expression of differentially expressed genes

In order to verify the expression of DEGs, quantitative real-time PCR was taken to measure the mRNA levels of the four DEGs. Total RNA was extracted from the tissue samples using TRIzol (Invitrogen). The RNA (1 µg) of each sample was used for reverse transcription with PrimeScript RT reagent (Takara). Then, PCR was performed using Power SYBR^®^ Green PCR Master Mix (TaKaRa), according to the manufacturer’s instructions. The primer sequences for quantitative PCR were *CNR1*: Forward 5′-TGTGCAGATGAAGGCTCAGG-3′; Reverse 5′-GAGCATTGGTACTGCCTGGT-3′. *PRKACB*: Forward 5′-AGGCTGGGATAACTAGCTTGA-3′; Reverse 5′- AAGCCCCTAGAAGCAAAGCA-3′. *CDKN3*: Forward 5′-GGACTCCTGACATAGCCAGC-3′; Reverse 5′-TGATGGTCTGTATTGCCCCG-3′. *PCLAF:* Forward 5′-ACATGGTGCGGACTAAAGCA-3′; Reverse 5′-AGGACATGCTCTITCCTCGAT-3′. *GAPDH:* Forward 5′-CGAAGGTGGAGTCAACGGATTT-3′; Reverse 5′- ATGGGTGGAATCATATTGGAAC-3′. Relative expression was normalized, and the 2^−ΔΔCt^ method was carried out to calculate the relative mRNA level, with GAPDH as an internal control.

### Immunohistochemistry and Western blot validation of the protein expression of differentially expressed genes

To verify the protein expression of differentially expressed genes, immunohistochemistry (IHC) and Western blot (WB) techniques were employed to detect the protein levels of four DEGs.

Immunohistochemistry: Paraffin-embedded tissue specimens were deparaffinized, dewaxed, and rehydrated in gradient ethanol. Sections were incubated with polyclonal rabbit anti-human antibodies diluted at 1:100 overnight at 4°C. CNR1, bs-1683R, Bioss; PRKACB, AF7746, Affinity Biosciences; CDKN3, DF6791, Affinity Biosciences; PCLAF, E-AB-52100, E-lab-science Biotechnology. The stained sections were scored by two independent pathologists who were blinded to the clinical outcomes. The total IHC scores for protein expression were semi-quantitatively calculated based on the positive cells and staining intensity. Scoring criteria: A: number of positive cells: 0%–1% = 0, 1%–10% = 1, 10%–50% = 2, 50%–80% = 3, 80%–100% = 4. B: staining intensity: 0 (Negative), 1 (Weakly positive), 2 (Positive), 3 (Strongly positive). Total scores = A × B.

Western blot: Tissues were solubilized in cell lysis buffer. After the total protein was extracted, the concentration was measured using the BCA assay (Thermo) method. Equivalent amounts of protein were separated by 12% SDS–PAGE and transferred into polyvinylidene fluoride (PVDF, Millipore) membranes. After being blocked by 5% bovine serum albumin (BSA) for 2 h at room temperature, the membranes were incubated with one of the following primary rabbit antibodies at 4°C overnight: *CNR1*, diluted at 1:1000, bs-1683R, Bioss; *PRKACB*, 1:1000, AF7746, Affinity Biosciences; *CDKN3*, 1:1000, DF6791, Affinity Biosciences; *PCLAF,* 1:1000, 81533, Cell Signaling Technology. GAPDH, diluted at 1:3000, 10494-1-AP, Proteintech Biotechnology. The membranes were washed three times with TBST, and the membrane was incubated with secondary anti-rabbit IgG (ZSGB-BIO) for 1.5 h. Finally, the bands were visualized using an ECL chemiluminescence kit, and the band intensity was quantified using ImageJ software.

### Statistical analysis

The statistical analysis was conducted using R version 4.2.2. If not specified, a Wilcoxon rank-sum test was applied for difference analysis inter-cohort with *p* < 0.05 as statistically significant.

## Results

### Candidate genes

**FIGURE 1:**
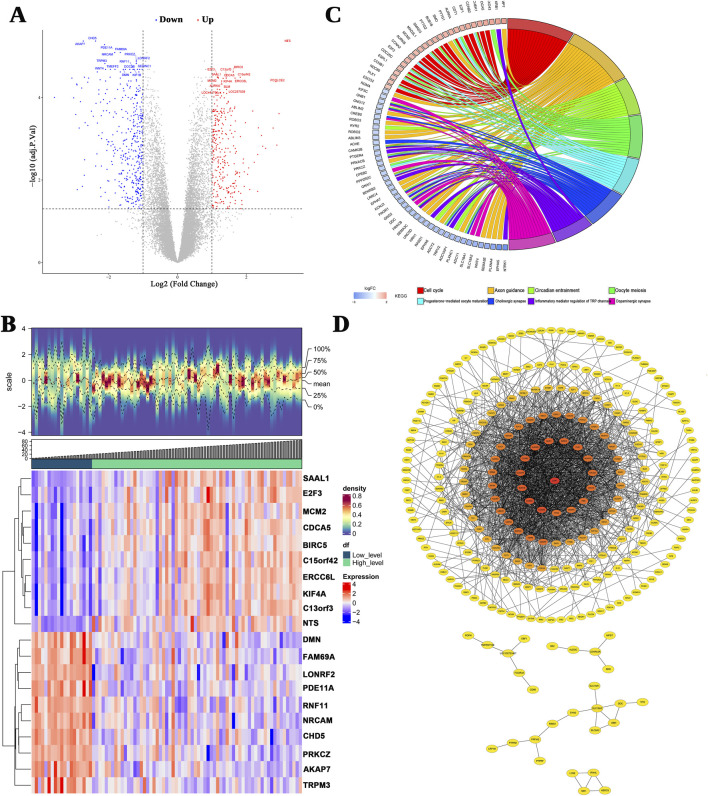
Differentially expressed genes (DEGs) between INSS early and advanced stages. **(A)** A volcano plot of the logFC and statistical significance of all DEGs. Red plots represent upregulated genes, and blue plots represent downregulated genes. Gray plots are genes that did not meet the criteria for DEGs. **(B)** The top 10 upregulated and downregulated genes are shown in this density heatmap. The expression levels of the genes are indicated by the colors in each cell (red for high and blue for low). **(C)** Top 10 significant KEGG signal pathways. **(D)** The PPI networks.

Of the 699 deferentially expressed genes (DEGs; Stage 1–2 versus Stage 3–4), 294 and 405 genes were upregulated and downregulated in the stages 1–2 and 3–4 groups, respectively (Figure 1A). The DEGs were ranked according to their adjusted *p*-values, and the top 10 up- and downregulated genes were selected for the density heatmap (Figure 1B). These DEGs were enriched in 389 gene ontology (GO) terms, including the regulation of “mitotic nuclear division,” “nuclear division,” and “mitotic nuclear division,” and 23 KEGG pathways, including the “cell cycle,” “axon guidance,” and “circadian entrainment” pathways (Figure 1C). The protein–protein interaction (PPI) network contained 268 nodes and 1,686 edges (Figure 1D). Furthermore, cluster1 (score = 36.550) contained 41 genes and 731 edges, cluster2 (score = 4.677) contained 10 genes and 21 edges, and cluster3 (score = 4.000) contained four genes and six edges (See Supplementary Figure S1). Thus, 55 genes were considered as candidate genes.

### Risk model to predict the survival of patients with NB

**FIGURE 2:**
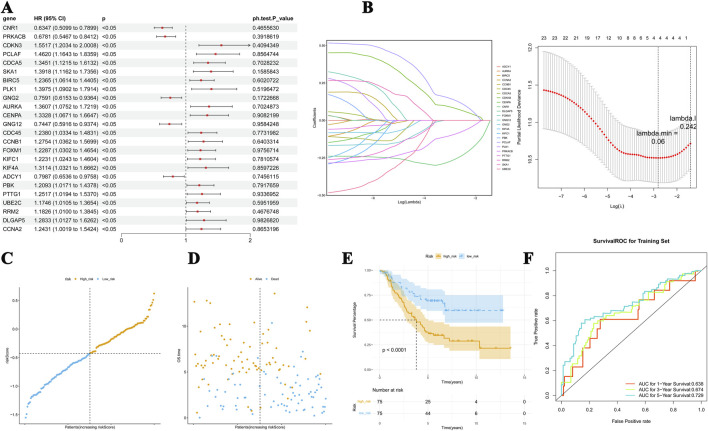
Risk model for predicting the survival status of NB. **(A)** Significance and hazard ratio values of stage-related DEGs in univariate Cox regression. All of the 55 candidate genes passed the PH test. **(B)** The LASSO model has the lowest error rate and yielded four prognostic genes, including *CDKN3*, *PCLAF*, *PRKACB*, and *CNR1*. **(C, D)** The distribution of risk scores, the association between risk scores and OS, and the mRNA expression of the four genes. **(E)** K–M curves showed dramatically higher concurrent survival rates of patients in the low-risk cohort than those in the high-risk group. **(F)** The ROC curve of the predictive performance of the four genes.

Using univariate Cox regression, 24 of the 55 candidate genes were screened for associations with prognosis, and all these genes passed the PH test (*p*-value >0.05, Figure 2A). The LASSO model with a lambda value minimum of 0.06 had the lowest model error rate and yielded four prognostic genes: *CNR1, PRKACB, CDKN3*, and *PCLAF* (Figure 2B). These four prognostic genes and their coefficients used to calculate the risk scores are listed in Supplementary Table S1. The patients in training set 1 were subgrouped as a high-risk cohort (n = 75) and a low-risk cohort (n = 75) using −0.426 as the cutoff value (Figures 2c, D). According to Kaplan–Meier (K–M) curves, the low-risk patients had considerably higher concurrent survival rates than high-risk patients (Figure 2E). The ROC curve demonstrates that the overall area under the curve (AUC) was above 0.600 (AUC values for 1 year, 3 years, and 5 years = 0.638, 0.674, and 0.729, respectively), suggesting that the risk scores could better predict the survival status of NB patients relatively accurately (Figure 2F).

**FIGURE 3:**
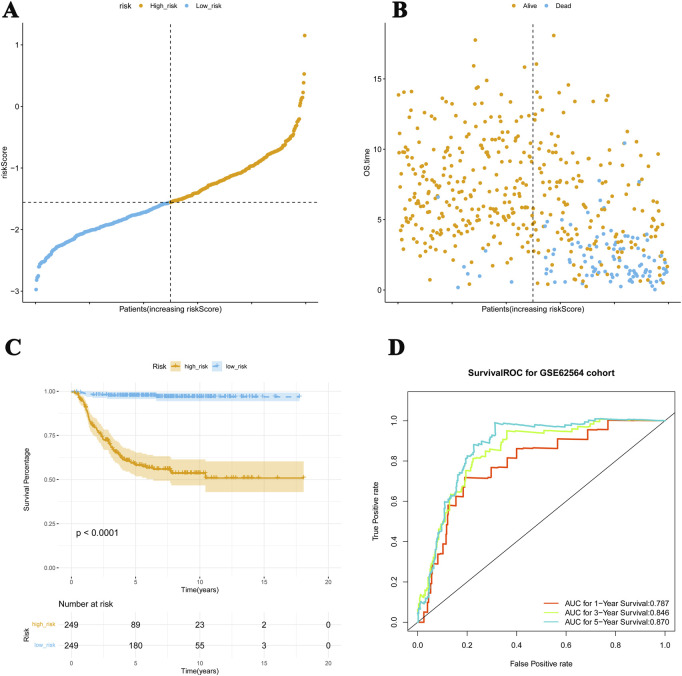
Risk model applied to the validation dataset. **(A, B)** For the validation set, patients are divided into high-risk and low-risk cohorts. **(C)** The K–M curves show meaningful differences in survival between these two cohorts (*p* < 0.05), which is consistent with the performance of the training set survival pattern. **(D)** The ROC curve verifies the predictive performance of these four genes.

For the validation set, the patients were divided into high-risk (n = 249) and low-risk (n = 249) cohorts using the median of the risk scores, −1.556 (Figures 3A, B). The K–M curves showed a significant difference in survival in these two cohorts (*p* < 0.05), and the results were consistent with the survival pattern and performance of the training set survival pattern (Figure 3C). In the ROC curve, the overall AUC values were also above 0.7 (Figure 3D), suggesting that the constructed risk model is applicable to different NB datasets.

### Predictive ability of the nomogram model

**FIGURE 4:**
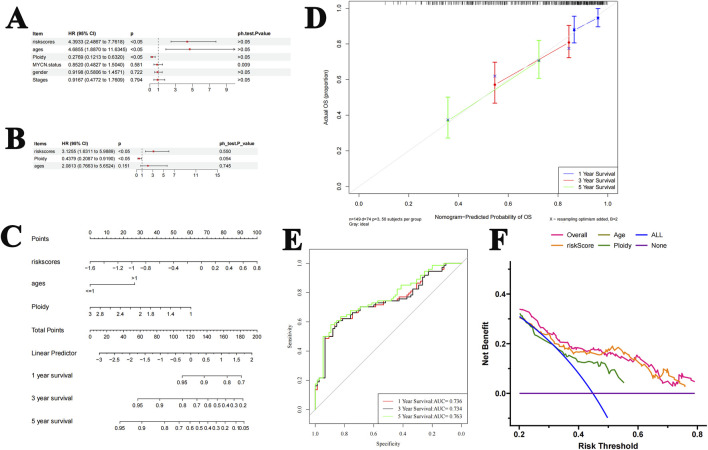
Construction and validation of the nomogram. **(A, B)**. Cox analysis shows that the risk score and ploidy are independent prognostic factors. **(C)** The nomogram model predicts the probability of patient survival at 1 year, 3 years, and 5 years. **(D)** The predictions for each year are all around the diagonal in the calibration curves. **(E)** The ROC curve showed that the AUC values for 1 year, 3 years, and 5 years were all greater than 0.7. **(F)** The DCA curve showed that the predictive effect of the nomogram was greater than that of other individual factors.

The predictive abilities of the risk score, age, gender, NB stages, ploidy, and MYC-N status were analyzed. The Cox regression analysis showed that risk score and ploidy were two good independent prognostic factors (Figures 4A, B). These two factors were, therefore, included in the construction of the nomogram. The nomogram model was developed using logistic regression based on independent prognostic factors. It allowed the prediction of the probability of patient survival after 1 year, 3 years, and 5 years based on the total number of points (Figure 4C). The predictions for the different periods were all close to the diagonal line in the calibration curves (Figure 4D). In the ROC curves, the AUCs were greater than 0.7 for all three periods (Figure 4E). Decision curve analysis showed that the predictive power of the nomogram was greater than that of the individual factors (Figure 4F). These results all reflect the good predictive ability of the nomogram model.

### Risk scores differed between the tumor stage, MYCN status, and age subgroups

**FIGURE 5:**
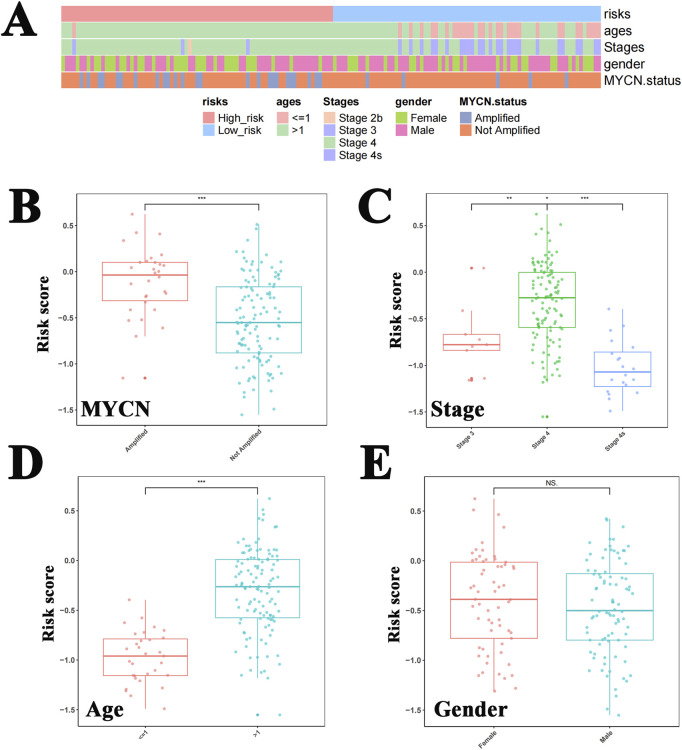
Risk scores differed between tumor stage, MYCN status, and age groups. **(A)** The heat map shows the distribution of clinical characteristics between the high-risk and low-risk cohorts. The risk scores differed significantly between the MYCN amplified status **(B)**, tumor stages **(C)**, and age subgroups **(D)**, but this significance was not observed between genders **(E)**.

A heat map was used to illustrate the distribution of clinical characteristics between the high- and low-risk cohorts (Figure 5A). The risk scores significantly differed between the different tumor stages, MYCN status, and age subgroups; however, no significant difference was observed between genders (Figures 5B–E).

### Major differential gene expression profiles

**FIGURE 6:**
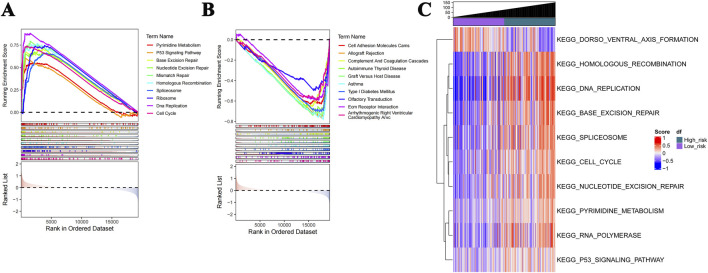
GSEA and GSVA profiles. **(A)** GSEA shows that the Top10 activated pathways in the high-risk group are closely related to the cell cycle, immune-inflammatory processes, and P53 signaling. **(B)** The Top10 pathways that are significantly inhibited. **(C)** The enriched pathways in GSVA are consistent with the GSEA results.

GSEA demonstrated enrichment in a total of 68 pathways closely related to the cell cycle, immune-inflammatory processes, and the p53 signaling pathways (Figure 6). Panel A represents the Top10 pathways that are significantly activated in the high-risk group; panel B represents the Top10 pathways that are significantly inhibited. Interestingly, the most significantly enriched pathways in the gene set variation analysis (GSVA) were consistent with the GSEA results (Figure 6C); they were mainly pathways closely related to the cell cycle and cell proliferation.

### Immune cells, immune checkpoint, and drug sensitivity profiles

**FIGURE 7:**
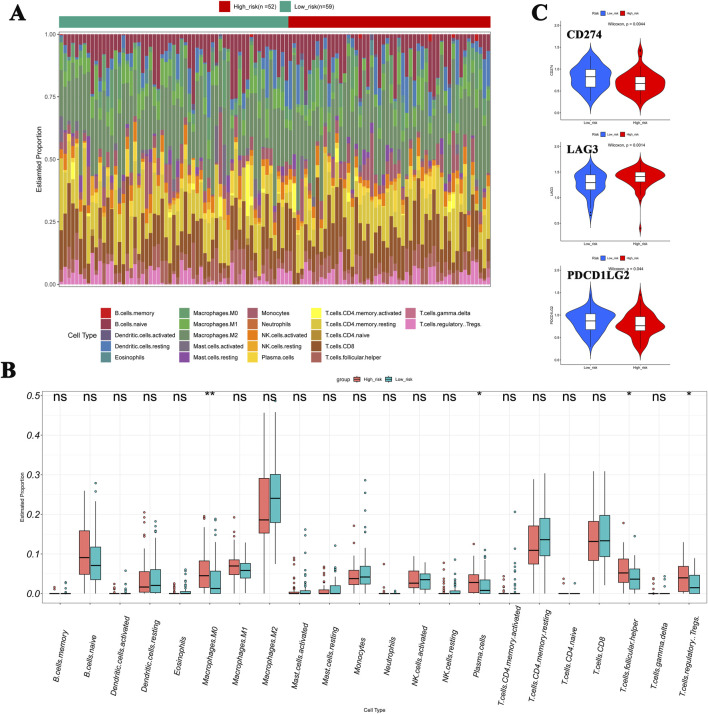
Immune cell infiltration in NB. **(A, B)** Compared to the low-risk cohort, the heat map showed that the proportions of M0 macrophages, plasma cells, follicular helper T cells, and Tregs were significantly higher in the high-risk cohort. **(C)** Expression levels of LAG3, CD274 (PD-L1), and PDCD1LG2 show significant differences between cohorts.

The heat map shows that the proportions of M0 macrophages, plasma cells, follicular helper T cells, and Tregs were markedly higher in the high-risk cohort than in the low-risk one (Figures 7A, B). In addition, the expression level of LAG3 was significantly higher in the high-risk group, while CD274 (PD-L1) and PDCD1LG2 were significantly downregulated (Figure 7C). By comparing the half-maximal inhibitory concentration (IC_50_) values of drugs between the risk cohorts, the top 20 drugs are identified and illustrated in Supplementary Figure S2.

### Verification of mRNA expression of DEGs in NB tissue samples

**FIGURE 8:**
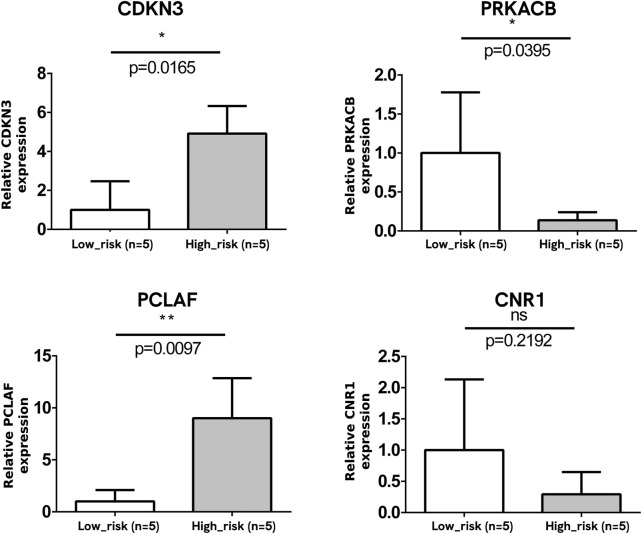
Verification of mRNA expression of DEGs. The mRNA expressions of CDKN3 and PCLAF were significantly upregulated and that of PRKACB was downregulated at the advanced stage. No significant change was observed in the *CNR1* mRNA level.

A total of 10 NB tumor samples were analyzed for the mRNA expression of four DEGs, with equal numbers of advanced-stage NB tissue samples (n = 5) and early-stage ones (n = 5). The mRNA expression levels of *CDKN3* and *PCLAF* were upregulated, and that of *PRKACB* was downregulated at the advanced stage, which was consistent with the bioinformatics analysis (*p* < 0.05, Figure 8). No significant change was observed in the *CNR1* mRNA level (*p* > 0.05).

### Verification of protein expression in NB tissue samples

**FIGURE 9:**
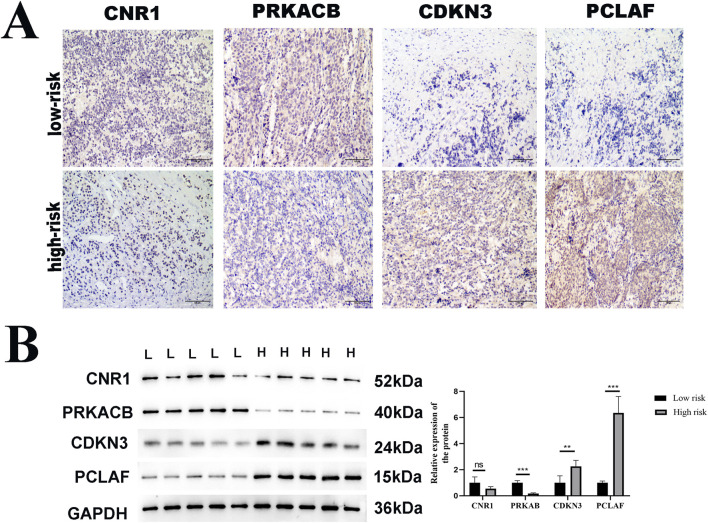
Verification of protein expression of DEGs. Immunohistochemistry (IHC) and Western blot (WB) analysis of *CDKN3*, *PCLAF*, *PRKACB*, and *CNR1* expression in low-risk versus high-risk NB tumor tissue samples. **(A)** Representative IHC images of these four genes in tumor tissues. Scale bar: 100 μm, **(B)** Western immunoblots. GAPDH was used to normalize. The expressions of CDKN3 and PCLAF were significantly upregulated and that of PRKACB was downregulated at the advanced stage of NB, compared with the early stage, with statistical significance indicated (*p < 0.05, **p < 0.01).

To clinically validate the four genes identified in our prognostic model, we conducted protein-level verifications, including immunohistochemistry (IHC) and WB. The clinical samples used for protein verification were the same as those used in the qPCR assay. The representative IHC images are illustrated in Figure 9A. The IHC (Figure 9A) and WB (Figure 9B) both show that the expressions of *CDKN3* and *PCLAF* in the advanced stages were significantly higher than in the early stages, while *PRKACB* was the opposite (*p* < 0.05). No significant change was observed in the protein level of *CNR1* (*p* > 0.05).

## Discussion

NB has an insidious onset without specific symptoms (Kembhavi et al., 2015), making early-stage diagnosis challenging. Approximately 50% of children with NB show metastases at initial diagnosis (Tolbert and Matthay, 2018), which contributes to a poor prognosis, especially in patients at an advanced INSS stage (Pizzo et al., 2015). The INSS has been an effective predictive tool for the prognosis of NB over the past decade in China (Brodeur et al., 1993). Patients with advanced INSS stages are resistant to current high-intensity therapy and typically have a poor prognosis compared to children in the early stages (Sokol and Desai, 2019; Maris et al., 2007). Researchers have speculated that this may be due to tumors that are more difficult to treat or that have spread to areas that are inaccessible for surgery; however, clinical evidence is lacking. By reviewing the clinical data of 72 patients with NB, Qi et al. found that advanced lung cancer inflammation index and INSS stage were independent prognostic factors for patients with NB (Qi et al., 2022). Keane et al. evaluated the expression of *DLG* and *LIN7* gene families in different INSS stages and found that the disrupted DLG isoform was abundant in the advanced stage of NB. This could control cell polarity and signaling, thereby affecting cancer cell viability (Keane et al., 2021). Further mechanistic studies investigating poor prognosis in children with advanced-stage NB are needed in the future.

In this study, we analyzed NB RNA-seq data from the TARGET and GEO databases to investigate the relationship between DEGs and NB prognosis. During screening, four genes (*CDKN3*, *PCLAF*, *PRKACB*, and *CNR1*) were identified as being significantly associated with advanced NB, and a risk model based on these four genes showed prognostic value for patient survival. These genes exert biological functions in the cell cycle, immunoinflammatory processes, and the p53 signaling pathway, and expression assays validated that the mRNA and protein levels of *CDKN3*, *PCLAF*, *PRKACB*, and *CNR1* in NB tissues correlate with NB staging.

As a member of the dual-specific protein phosphatase family and kinase-associated phosphatases, the cyclin-dependent kinase inhibitor 3 (*CDKN3*) gene product inhibits the G (1)/S transition by dephosphorylating cyclin-dependent kinases (Ding et al., 2020). In our report, we observed that the level of *CDKN3* was elevated in the advanced-stage NB. Several recent studies have reported that *CDKN3* plays a regulatory role in the survival and proliferation of cancer cells. Yang et al. found that *CDKN3* is a marker of poor prognosis in NB (Yang et al., 2017). Vernaza et al. found that knocking down *CDKN3* reduced the expression of the cell proliferation markers Ki67 and proliferating cell nuclear antigen (PCNA), a binding partner of PCLAF, and decreased the colony formation of NB cells (Vernaza et al., 2024). The *in vitro* functional assays have been conducted in liver hepatocellular carcinoma (LIHC). Q-PCR and Western blotting were used to detect the expression of *CDKN3* in LIHC and its adjacent tissues as well as human liver cancer cell lines. It was found that, compared with the adjacent samples, the mRNA expression of the *CDKN3* gene was significantly upregulated in 63.9% (23/36) of liver cancer tissues. After silencing the expression of *CDKN3*, the growth and colony formation ability of cancer cells were significantly inhibited (Li et al., 2023). Moreover, silencing the expression of *CDKN3* could induce G0/G1 phase arrest in liver cancer cells, indicating that *CDKN3* promotes the proliferation of liver cancer cells by affecting the cell cycle distribution (Lin et al., 2013). Knockdown of *CDKN3* led to the downregulation of p53 and p21 protein levels, while the AKT serine/threonine kinase 1 was upregulated (Dai et al., 2016). Therefore, the elevated expression of *CDKN3* may reduce the survival of tumor cells and change the sensitivity to therapeutic agents through the AKT/P53/P21 signaling pathway (Dai et al., 2016). These observations facilitate translating genomic and transcriptomic features into molecular mechanisms of high-risk NB.

Proliferating cell nuclear antigen-clamp associated factor (*PCLAF*) is involved in DNA repair through its binding partner, proliferating cell nuclear antigen (*PCNA*) (Liu et al., 2021). *PCLAF* has been reported to play various essential roles in cell proliferation, apoptosis, and cell cycle regulation (Liu et al., 2021). Among the differentially expressed downregulated genes in NB, Liu et al. found that the cell cycle-related pathway genes were most significantly altered in association with the decreased level of *PCLAF* (Liu et al., 2022a). The patients with elevated levels of *PCLAF* mRNA had the worst OS and EFS, which was consistent with our results. Importantly, *PCLAF* knockdown could limit the proliferation of NB cells *in vitro* and *in vivo* (Liu et al., 2022a). In eight types of tumors, including cholangiocarcinoma, cervical cancer, and glioblastoma multiforme, the expression levels of *PCLAF* are significantly higher than those in normal tissues (Liu et al., 2022b). Meanwhile, the DNA methylation level of *PCLAF* is decreased, indicating an association between hypomethylation and high expression (Liu et al., 2022b). The survival analysis conducted using the GEPIA2 tool showed that the high expression of the *PCLAF* gene is correlated with the overall survival and disease-free survival of patients, suggesting that it can serve as a potential prognostic biomarker. At the same time, this pan-cancer study also found that the expression of *PCLAF* is positively correlated with activated CD4^+^ T cells and T helper 2 (Th2) cells. This implies that *PCLAF* may play a specific role in the process of tumor immune cell infiltration and affect the functions and distribution of immune cells in the microenvironment (Liu et al., 2022b). These results, together with ours, reveal the mechanism through which *PCLAF* facilitates cell cycle progression, suggesting this gene may be a therapeutic target in NB.

Protein kinase cAMP-dependent catalytic subunit beta (PRKACB) is a member of the serine/threonine protein kinase family and a key effector of cAMP/PKA-induced signal transduction (Soberg et al., 2017). It is involved in numerous cellular processes, including cell proliferation, apoptosis, gene transcription, metabolism, and differentiation (Ahmed et al., 2022; Huang et al., 2024). Shao et al. found significantly lower *PRKACB* expression in an N-myc proto-oncogene (MYCN)-amplified group than in an MYCN-non-amplified group (Shao et al., 2021). In the present study, *PRKACB* downregulation was observed in children with poor OS rates. Whole genome-scale integrated analyses of exon arrays demonstrated that *PRKACB* is a novel cancer-related variant transcript in gastric cancers (Furuta et al., 2012). The expression of *PRKACB* was downregulated in both colorectal cancer and non-small cell lung cancer (NSCLC) (Yao et al., 2020; Chen et al., 2013). In a study aiming to evaluate the expression pattern of *PRKACB* in NSCLC, Chen et al. found that *PRKACB* is downregulated in NSCLC tissues, and the upregulation of this gene decreased the number of proliferative, colony-forming cells and invasive cells, indicating that an upregulated *PRKACB* level may be an effective way to prevent the progression of NSCLC (Chen et al., 2013). In a large-scale study in 2023 involving a nutrient database and cellular and animal assays, trans-vaccenic acid (TVA) was found to lead to an increased *PRKACB* expression in immunodeficient nude mice, T cell receptor-α knockout mice, and mice with a CD8^+^ T cell depletion. When the gene was upregulated, the cAMP-PKA-CREB axis was activated, which not only enhanced the function of CD8^+^ T cells but also reversed T cell exhaustion (Fan et al., 2023). This multi-faceted approach provides a new potential direction; that is, the anti-tumor effect could be enhanced by regulating the intake of related nutrients based on their gene regulation mechanisms.

Cannabinoid receptor-1 (*CNR1*) is a classical receptor of the endocannabinoid system that primarily exists in the central and peripheral nervous systems (Tao et al., 2020). Depending on its binding to its corresponding ligands, *CNR1* activates intracellular signaling pathways related to survival and is a potential biomarker of poor prognosis in cancer (Zou and Kumar 2018). In recent years, cannabinoids (CB) have received increasing attention due to their effects on cancer growth. CB2 agonists (JWH-015; JWH-133) have been proved to reduce the size of breast cancer tumors (Blanton et al., 2022). In triple-negative breast cancer (TNBC), *CNR1* regulates fatty acid metabolism by modulating the PI3K-AKT/MAPK signaling pathways, thereby affecting the sensitivity of cells toward ferroptosis (Li et al., 2022). Moreover, the combined use of *CNR1* antagonists and ferroptosis inducers has demonstrated good anti-tumor effects in TNBC (Li et al., 2022). In studies on the association between liver metabolism and tumors, the activation of *CNR1* can specifically induce the expression of the *CREBH* gene through the phosphorylation of the JNK signaling pathway and the binding of c-Jun to the AP-1 in the promoter of the *CREBH* and then regulating hepatic gluconeogenesis (Chanda et al., 2011). In neuroblastoma, this signaling pathway may also be involved in regulating the proliferation, metabolism, and other processes of tumor cells. Mu et al. compared 89 DEGs between MYCN amplification and non-amplification groups and suggested that *CNR1* plays a prognostic role in NB, although they did not perform any verification experiments (Schmitt et al., 1986). Decock et al. applied two independent genome-wide methylation screening methods to eight NB cell lines and found that *CNR1* methylation was associated with classical risk factors, including stage and MYCN status, but not with OS, EFS, or both (Decock et al., 2012). Although the bioinformatic analysis revealed an association between the NB stage and *CNR1*, remarkably different *CNR1* expression levels were not observed in the clinical samples in our study. This may have been owing to tumor heterogeneity or the small sample size. Thus, further analyses are required to clarify the role of *CNR1* as an independent prognostic biomarker.

MYCN gene amplification (defined as a copy number greater than 10-fold) is an internationally recognized molecular event in high-risk NB, and this alteration is present in approximately 20%–30% of cases (Qiu and Matthay, 2022). Clinical data indicate that the 5-year survival rate of patients with MYCN gene amplification is significantly lower than that of patients without it (Schwalbe et al., 2017). We used the predictive model constructed from the four DEGs to divide patients into high-risk and low-risk groups. We found that the positive amplification rate of the MYCN gene in the high-risk group was significantly higher than that in the low-risk one. This confirmed that this risk model was significantly correlated with the MYCN molecular type.

The chromosomal ploidy of NB is classified into near-diploid (Diploid) and near-triploid (Triploid). Near-diploid tumors are often accompanied by deletions or gains of chromosomal segments and are associated with MYCN amplification and a high risk of recurrence (Irwin et al., 2021). Near-triploid tumors are more common in infants, mostly low-risk babies, and may resolve spontaneously. Studies have shown that near-diploid tumors have higher genomic instability and are prone to accumulating MYCN amplification, leading to treatment resistance and recurrence (Benard et al., 2008). In this study, through performing Cox, ROC, and DCA analysis, we found that ploidy has independent prognostic value and can provide a more reliable basis for clinical decision-making in NB. Currently, the International Neuroblastoma Risk Group (INRG) system has integrated factors such as MYCN amplification, patient age, tumor stage, and ploidy to guide NB treatment. Intervention strategies targeting the MYCN gene include the following three aspects: (i) directly interfering with MYCN transcription, for instance, using peroxisome proliferator-activated receptor γ (PPAR-γ) antagonists to induce cell differentiation by inhibiting MYCN expression (Nakao-Ise et al., 2023); (ii) metabolic intervention: aldehyde dehydrogenase 18 family member A1 (ALDH18A1) inhibitors can block the MYCN-metabolic feedback loop and have shown significant efficacy in animal models (Guo et al., 2020); (iii) epigenetic regulation: histone deacetylase inhibitors or DNA methyltransferase inhibitors can reverse the epigenetic silencing driven by MYCN (Eppet et al., 2023). Due to the synergistic effect between ploidy and MYCN amplification (Park et al., 2012), multi-omics integration analysis, as well as studies combining MYCN inhibitors with immune checkpoint inhibitors (ICIs), have shown therapeutic potential in long-term follow-up (van Tilburg et al., 2020). In the future, multi-dimensional molecular stratification and targeted intervention will be applied to improve the survival outcomes of high-risk patients.

Immunotherapy has been proved to be an effective treatment for solid tumors in adults (Anderson et al., 2022). However, evaluations of tumor mutational burden have shown that neuroblastoma ranks first among human cancers with the fewest mutations, and it also has the lowest infiltration of immune cells (Anderson et al., 2022). Therefore, due to the “cold” state of NB in terms of immunity and mutation, its immunotherapy seems to have reached a bottleneck (Grobner et al., 2018). Fortunately, the inherent immune evasion mechanism of neuroblastoma is similar to that of adult tumors (Wienke et al., 2021). Anderson et al. mentioned that the immunotherapy of NB should not only focus on increasing tumor antigenicity; addressing the problem of the tumor immunosuppressive microenvironment is also crucial (Anderson et al., 2022).

In the present work, after evaluating the infiltration of immune cells in high- and low-risk patients using the established model, we observed that the infiltration of immune cells in these two groups was different: the numbers of macrophage M0, plasma cells, follicular helper T cells, and Tregs in the high-risk cohort were significantly higher than in the low-risk group. Research by Zhang et al. showed that the infiltration degree of M0 macrophages in hepatocellular carcinoma tissues was remarkably higher than that in normal liver tissues (Zhang et al., 2022), which was consistent with our results. Together with our findings, these results indicate that macrophage infiltration is related to poor prognosis, and immunotherapy may be effective because macrophages can enhance phagocytosis of tumors in high-risk NB. M2 macrophages, which are polarized from M0 macrophages, can release cytokines and chemokines such as transforming growth factor-β (TGF-β), vascular endothelial growth factor (VEGF), and indoleamine-2,3-dioxygenase (IDO). These inhibitory factors substances could suppress the anti-tumor immune response (Iolascon et al., 2000). Meanwhile, these substances will recruit plasma cells and Tregs into the tumor microenvironment, where they directly kill effector T cells or inhibit their functions and facilitate tumor cells escaping immune surveillance (Anderson et al., 2022). Kang et al. reported that the proportion of follicular helper T cells was higher in the high-risk cohorts than in the low-risk NB group (Kang et al., 2021). Mei et al. used single-cell RNA-seq to conduct a comparative analysis of patients with and without metastasis and observed macrophage enrichment, T-cell exhaustion, and increased numbers of Tregs (Mei et al., 2024). Methods need to be found to inhibit the functions or reduce the number of regulatory T cells to break the immunosuppressive state. However, relevant research is still under exploration (Anderson et al., 2022; Zhu et al., 2023).

Cancer immunotherapy has achieved remarkable success due to the application of immune checkpoint inhibitors (ICI) in the treatment of adult tumors and pediatric hematological malignancies (Davis et al., 2017). However, in contrast, most children with solid tumors, including high-risk neuroblastoma, have hardly benefited from ICI (Anderson et al., 2022). Using the model we established, the patients were divided into high- and low-risk groups. We found that the upregulated lymphocyte activation gene 3 (*LAG3*) was significantly upregulated in the high-risk cohort. It has been observed that the *LAG3* gene exerts an inhibitory function on antigen-activated T cells (Camisaschi et al., 2010). In addition to *LAG3*, two other important checkpoint molecules expressed by tumor cells, namely, the PD-L1 ligand (CD274) and its paralog PDCD1LG2, were significantly decreased in the high-risk groups. By suppressing the expression of PD-L1, tumor cells weaken the signals transmitted to immune cells, thus facilitating tumor escape (Mezzadra et al., 2017). Tang et al. studied the mechanism of PD-1 blockade therapy and found that macrophages are important effector cells in anti-high-risk NB immunity (Tang et al., 2022). PD-1 blockade can lead to the exhaustion of macrophages, a decrease in their phagocytic potency, a reduction in tumor growth, and an increase in survival in mouse models of NB in a macrophage-dependent manner (Gordon et al., 2017). Recent studies have focused on optimizing existing GD2 antibodies, developing novel bispecific antibodies or chimeric antigen receptor T cell (CAR-T) therapies, and exploring the combined application of ICI (NCT05437315, NCT02914405, etc.). Although the efficacy of a single ICI is limited, combined regimens (such as anti-GD2 + anti-PD-1) and novel immunomodulatory strategies are showing potential in clinical application (Kennedy et al., 2023). Immunotherapy combined with chemotherapy has been added to the standard treatment regimen recommended for relapsed or refractory neuroblastoma (Anderson et al., 2022). It will be necessary to verify the safety and long-term efficacy of these therapies through clinical trials. The findings related to immune cell infiltration and checkpoints in this study are expected to serve as targets for drug development, aiming to restore the functions of immune cells that have been damaged during chemotherapy.

## Limitations

This study had several limitations. First, the main sources of clinical information, the online databases, are limited and incomplete; some biological factors, such as individual therapeutic regimens, cannot be obtained. Second, limited by the current research conditions, the verification of the biological functions was only performed *in vivo*. It is recommended that in further studies, the cancer-promoting mechanisms of these genes should be preferentially verified *in vitro*.

## Conclusion

Our study proposes an INSS stage-related risk model that combines genetic and clinical characteristics to predict the prognosis of patients with NB. Four prognostic genes (*CNR1, PRKACB, CDKN3,* and *PCLAF*) were identified, and a prognostic model based on these genes was developed that provides novel insights into the prognostic evaluation of neuroblastoma. In clinical applications, the diversity of biomarkers and their implication on clinical features can facilitate the formation of targeted therapeutic strategies and the development of individualized immunotherapy regimens.

## Data Availability

The raw data supporting the conclusions of this article will be made available by the authors, without undue reservation.

## Supplementary material

SUPPLEMENTARY FIGURE S1 The PPI network showed a total of 55 genes were considered as candidate genes in the three clusters.

SUPPLEMENTARY FIGURE S2 The top 20 drugs include AS605240_224, AST_1306_381, AZD7762_1022 et al.

## Abbreviations

ALI, advanced lung cancer inflammation index; AUC, area under the curve; CDKN3, cyclin-dependent kinase inhibitor 3; COG, Children’s Oncology Group; CNR1, cannabinoid receptor-1; DEG, differentially expressed gene; GEO, Gene Expression Omnibus; INSS, International Neuroblastoma Staging System; KAP, kinase-associated phosphatase; LASSO, least absolute shrinkage and selection operator; NB, neuroblastoma; PCLAF, proliferating cell nuclear antigen-clamp associated factor; PCNA, proliferating cell nuclear antigen; PPI, protein–protein interaction; PRKACB, protein kinase cAMP-dependent catalytic subunit β; ROC, receiver operating characteristic; TARGET, Therapeutically Applicable Research to Generate Effective Treatments; LASSO, least absolute shrinkage and selection operator.
