# Supplementary figures and images for: Identification and verification of international neuroblastoma staging system (INSS) stage-related genes as potential biomarkers for neuroblastoma prognostic models
Source: Front Cell Dev Biol. 2025 Apr 15;13:1502380. doi: 10.3389/fcell.2025.1502380 (PMC12037508; doi:10.3389/fcell.2025.1502380)

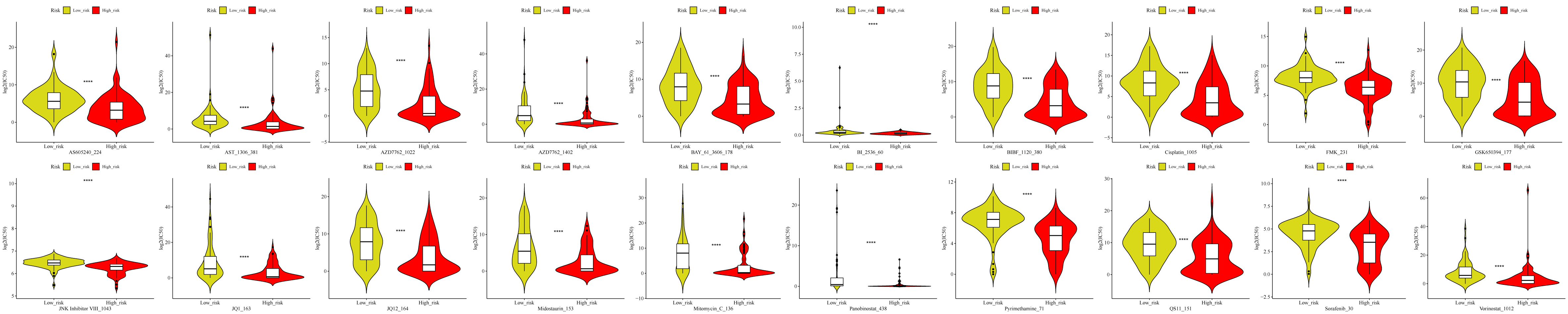

Supplement: Supplementary file 1 [file Image2.tif]

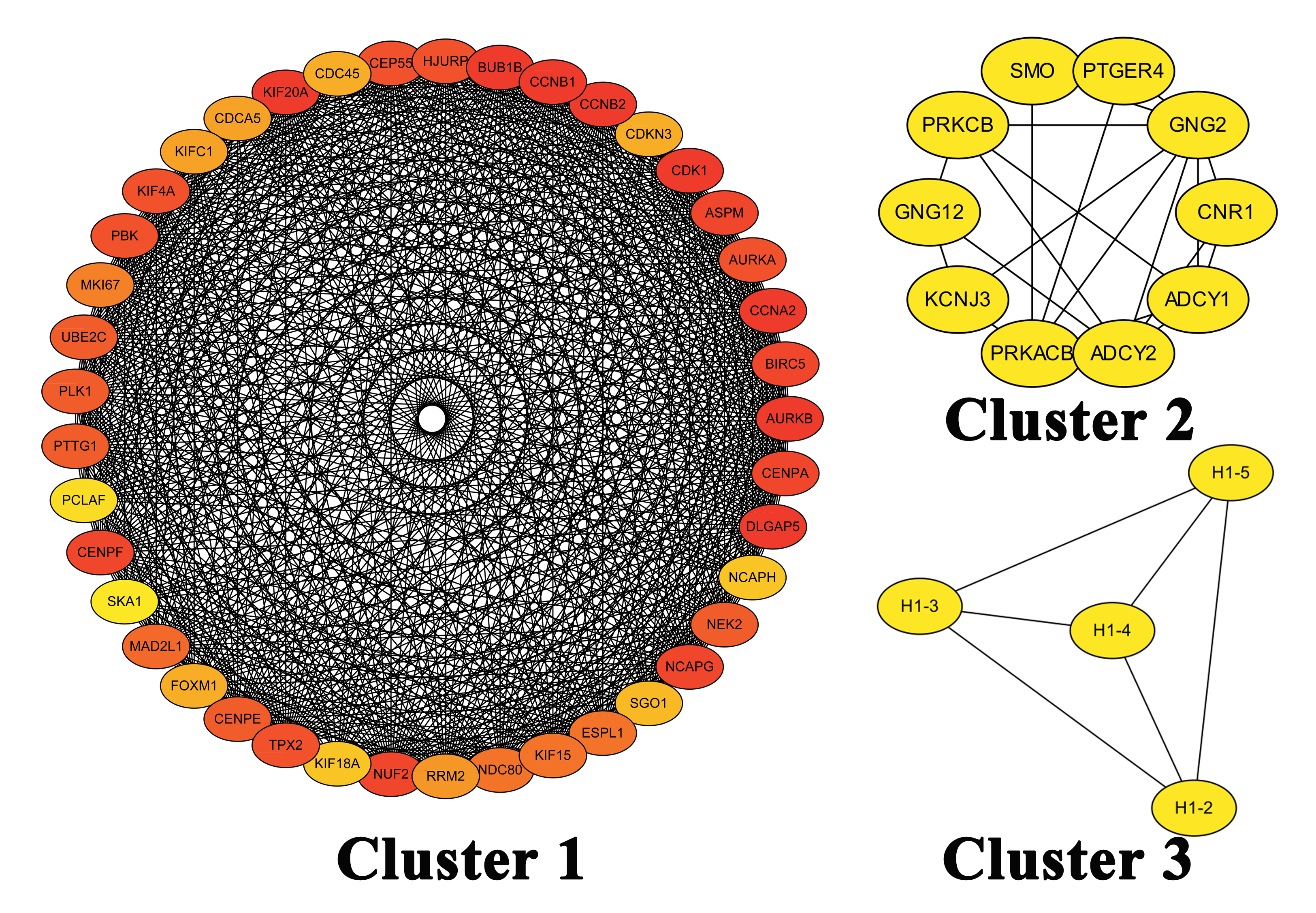

Supplement: Supplementary file 2 [file Image1.tif]
